# Conceptual framework for neuronal ensemble identification and manipulation related to behavior using calcium imaging

**DOI:** 10.1117/1.NPh.9.4.041403

**Published:** 2022-07-25

**Authors:** Luis Carrillo-Reid, Vladimir Calderon

**Affiliations:** National Autonomous University of Mexico, Neurobiology Institute, Department of Developmental Neurobiology and Neurophysiology, Querétaro, Mexico

**Keywords:** neuronal ensembles, dimensionality reduction, graphical methods, two-photon imaging, two-photon optogenetics, population vectors

## Abstract

**Significance:**

The identification and manipulation of spatially identified neuronal ensembles with optical methods have been recently used to prove the causal link between neuronal ensemble activity and learned behaviors. However, the standardization of a conceptual framework to identify and manipulate neuronal ensembles from calcium imaging recordings is still lacking.

**Aim:**

We propose a conceptual framework for the identification and manipulation of neuronal ensembles using simultaneous calcium imaging and two-photon optogenetics in behaving mice.

**Approach:**

We review the computational approaches that have been used to identify and manipulate neuronal ensembles with single cell resolution during behavior in different brain regions using all-optical methods.

**Results:**

We proposed three steps as a conceptual framework that could be applied to calcium imaging recordings to identify and manipulate neuronal ensembles in behaving mice: (1) transformation of calcium transients into binary arrays; (2) identification of neuronal ensembles as similar population vectors; and (3) targeting of neuronal ensemble members that significantly impact behavioral performance.

**Conclusions:**

The use of simultaneous two-photon calcium imaging and two-photon optogenetics allowed for the experimental demonstration of the causal relation of population activity and learned behaviors. The standardization of analytical tools to identify and manipulate neuronal ensembles could accelerate interventional experiments aiming to reprogram the brain in normal and pathological conditions.

## 1 Introduction

Neuroscience experiments aiming to causally relate learned behaviors to the activity of neurons require the identification and manipulation of neuronal ensembles with high spatial resolution.^1^^–^^3^ Recently, the use of simultaneous two-photon calcium imaging and two-photon optogenetics demonstrated that the activation of neuronal ensembles with nearly single cell resolution can evoke learned behaviors in mice.^4^^–^^8^ In this context, a neuronal ensemble could be simply defined as a group of neurons with coordinated activity that can trigger the execution of a learned behavior.^9^ The idea that a group of neurons with recurrent activity could represent the basic module of brain computations was first proposed decades ago by Lorente de Nó.^10^ Years later, Hebb postulated that groups of neurons that fire together could increase their connectivity giving rise to “cell assemblies.”^11^ Even though Lorente de Nó’s and Hebb’s postulates have been fundamental for neuroscience studies, the ultimate definition of “neuronal ensembles” is still lacking in neuroscientific literature^9^ because different definitions are biased by the experimental techniques used. Accordingly, there could be several approximations to define what a neuronal ensemble is. (1) From the electrophysiological point of view, it has been proposed that neuronal ensembles are groups of neurons with synchronous activity^12^ with high probability to have direct synaptic connections.^13^ (2) From the calcium imaging point of view, neuronal ensembles are groups of neurons with concomitant activity that represent similar features of sensory stimuli,^14^ movements,^15^ contextual memories,^16^ spatial maps,^6^ short-term memory,^8^ or social interactions.^7^ (3) From the anatomical point of view, neuronal ensembles are groups of neurons spatially and functionally organized that increased their activity across different brain areas.^17^ (4) From the theoretical point of view, neuronal ensembles are attractor points in dynamical systems.^18^^,^^19^ In this review, we focus on all-optical interventional experiments using calcium imaging that, due to technical limitations, cannot consider the anatomical arrangement of ensemble members in different brain areas, their synaptic connectivity, or a fine description of their temporal dynamics. All of these considerations of an ultimate definition of what a neuronal ensemble is require the further development of high spatial and high temporal resolution methods that are beyond the scope of this review.^20^

We present a conceptual framework based on recent experiments combining calcium imaging and two-photon optogenetics that were used to identify and manipulate neuronal ensembles with single cell resolution to manipulate learned behaviors.^4^^–^^8^

**Fig. 1:**
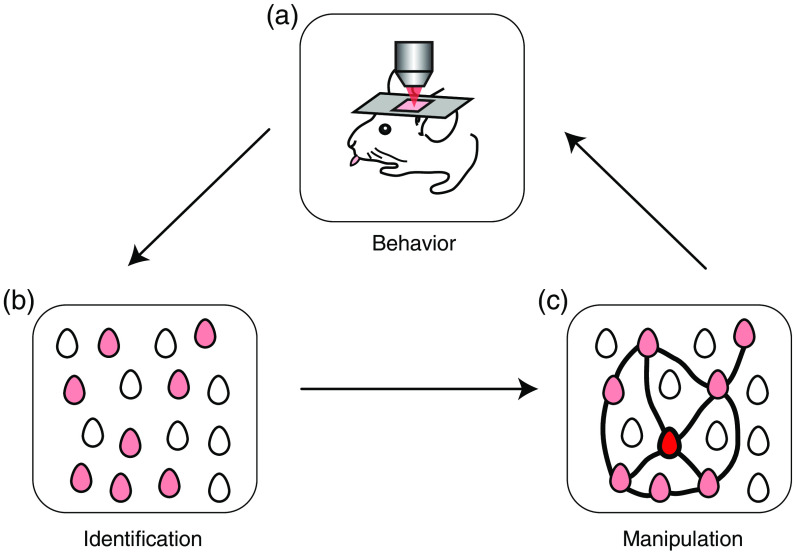
Interventional experiments in behaving mice. (a) Behavioral training and recording of the brain area related to the task. (b) Identification of neuronal ensembles associated with the correct execution of the learned task. (c) Manipulation of neuronal ensembles relevant to behavior.

Interventional experiments aiming to control learned behaviors in mice can be summarized as follows: (1) implementation of a behavioral task and an optical window to the brain region related to such behavior; (2) recording of population activity with high spatial resolution to identify neurons related to the correct execution of the learned task; (3) manipulation of targeted neurons that can recall task-related neuronal ensembles; and (4) assessment of task performance due to activation of targeted neurons (Fig. 1).

Several papers have reviewed in detail the microscope implementation for simultaneous two-photon calcium imaging and two-photon optogenetics,^2^^,^^3^^,^^21^^,^^22^ the automatic identification of neurons,^23^^,^^24^ the extraction of spikes from calcium transients,^25^^,^^26^ the detection of neuronal ensembles from calcium imaging recordings,^27^^,^^28^ and the methodological steps to perform interventional experiments in behaving mice.^29^ Therefore, in this review, we focus on a practical conceptual framework for the identification and manipulation of neuronal ensembles related to behavior.

As with any technique used in neuroscience, calcium imaging has advantages and disadvantages. The main advantage for interventional experiments is the high spatial resolution^30^^,^^31^ that allows for the long-term recording of the same field of view^32^ to identify and target selected neurons related to behavior.^4^^–^^7^ The main disadvantage of calcium imaging recordings is the low temporal resolution that limits the interpretation of recorded data in terms of high temporal resolution trajectories, dynamical systems, or population codes.^33^

Previous experiments have demonstrated that the activation of a single neuron rather than a group of neurons could evoke some behavioral readout,^34^^,^^35^ but after several years, such consequences were attributed to the reactivation of neuronal ensembles triggered by a single neuron.^36^^,^^37^

**Fig. 2:**
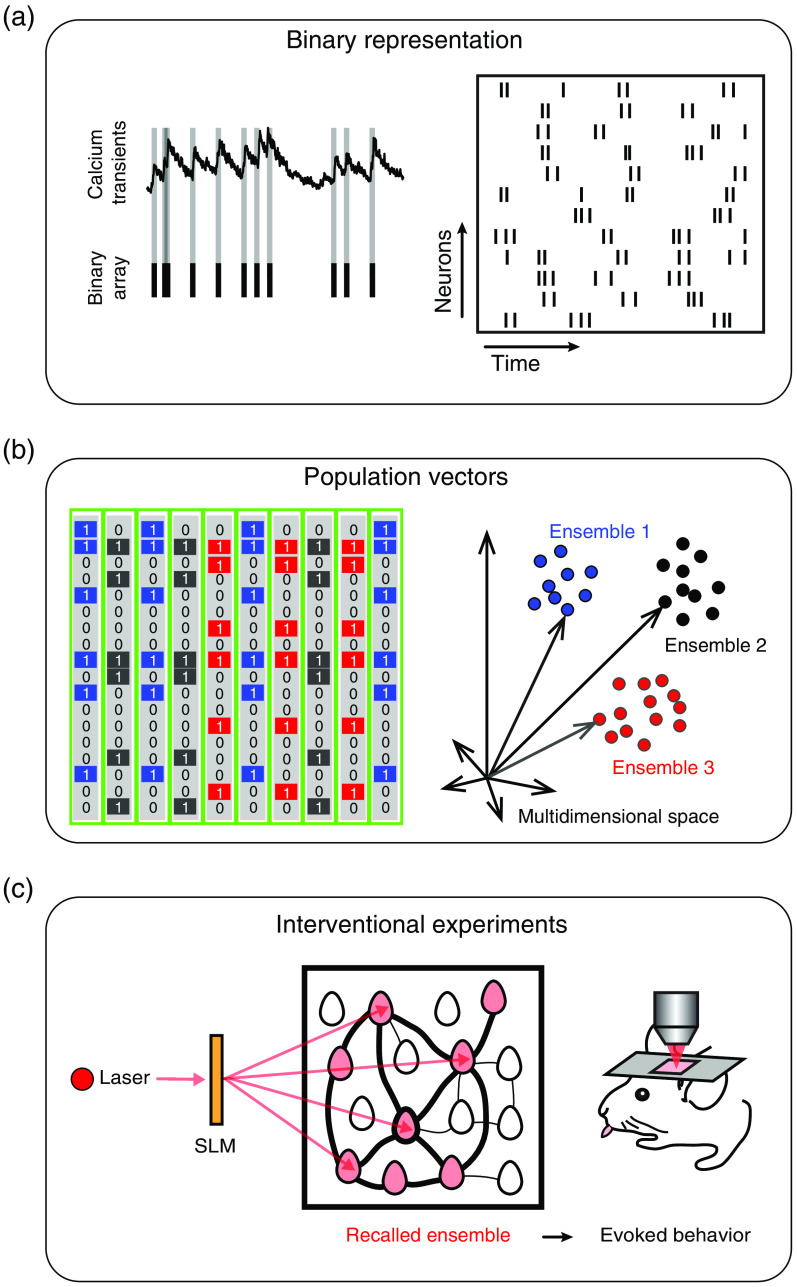
Conceptual framework for neuronal ensemble identification and manipulation. (a) Left: Transformation of calcium transients into binary arrays. Right: Binary representation of population activity, where rows represent neurons and columns represent time windows. (b) Left: Population vectors extracted from binary arrays. Right: Multidimensional representation of population vectors. Each dot depicts a population vector. Each cluster defines a neuronal ensemble that represents similar groups of neurons with coordinated activity at different times. (c) Interventional experiments using holographic two-photon optogenetics to target and recall neuronal ensembles relevant to behavior.

In this review, we propose three steps as a conceptual framework for interventional experiments during behavior: (1) transformation of calcium transients into binary arrays; (2) identification of neuronal ensembles as similar population vectors; and (3) targeting of neuronal ensemble members that significantly impact behavioral performance (Fig. 2).

The goal of this review is to propose a conceptual framework for the identification and manipulation of neuronal ensembles related to learned behaviors using simultaneous two-photon calcium imaging and two-photon optogenetics. In the next sections, we describe the main steps of this conceptual framework and the restrictions and considerations to identify and manipulate neuronal ensembles from calcium imaging recordings during behavior.

## 2 Transformation of Calcium Signals into Binary Arrays for Neuronal Ensemble Identification in Behaving Mice

Even though last generation genetically encoded calcium indicators can report a single action potential^38^^,^^39^ and two-photon optogenetics could have single spike precision,^2^^,^^3^^,^^20^ neuronal ensemble identification for interventional experiments in behaving mice until now have used bursts of action potentials and have not considered spike rates or synchrony with single spike precision.^4^^–^^7^ Despite the fact that spike rates have been proposed as the underlying mechanism for several brain computations,^40^ spike inference from calcium transients is not a trivial transformation.^26^ Inferred spikes from optical recordings are limited by the sampling rate, the expression levels of calcium indicators, and possibly being different in different cells.^41^ It has been recently suggested that the use of nonlinear models to infer spikes from calcium transients could yield results that resemble electrophysiological data;^33^ however, the limitation of the low sampling rate from scanning microscopy used for *in vivo* experiments stills represents an unavoidable limitation to design interventional experiments in behaving mice with single spike synchrony. Recently, it has been demonstrated that spike inference from calcium transients compared with electrical recordings produced different interpretations of population analyses and individual neuron properties, highlighting that spike inference should be cautiously considered at least for interventional experiments in behaving mice.^33^ On the other hand, it has been proposed that the total spike count in a brief time window (100 ms) independent of the spike frequency can guide behavior,^42^ indicating that bursting activity could be sufficient for interventional experiments in behaving mice. Thus, because bursting activity represents a robust measurement that can be extracted from calcium transients, we highlight the importance of the rising phase of calcium transients for neuronal ensemble identification and manipulation [Fig. 2(a)].^14^^,^^43^ Therefore, a robust approach for neuronal ensemble identification requires the preprocessing of raw calcium transients to reflect bursts of action potentials. Simultaneous electrophysiological and calcium imaging recordings demonstrated that detection of the positive slopes of the first-time derivative from filtered calcium transients is sufficient for detecting bursting activity.^44^^,^^45^ Time intervals of fluorescence rises evoked by bursting activity can be represented by ones and the absence of bursting activity by zeros.^4^^,^^45^^,^^46^ The transformation of the first-time derivative into binary arrays requires a hard threshold procedure that is usually determined by simultaneous imaging and electrophysiological recordings *in vivo* in the same experimental conditions in which interventional experiments are performed. A high threshold could originate sparse population activity requiring more trials for the identification of neuronal ensembles, whereas a low threshold could make ensemble identification challenging due to spurious correlations. The main advantages of the binary representation of population activity proposed here are the reduction of processing times^27^^,^^43^ and the elimination of artifacts caused by the decreasing phase of raw florescence signals,^14^^,^^27^ such advantages are fundamental for interventional experiments in which animals are engaged in the task for a limited time.

## 3 Identification of Neuronal Ensembles as Similar Population Vectors

The bursting activity of recorded neurons could be visualized as a binary matrix of size N (neurons) × T (time).^44^^,^^47^ From such a matrix, neuronal ensembles could be understood as population vectors that lie in “N” dimensions, where the number of dimensions represents the number of recorded neurons^44^^,^^46^^,^^47^ [Fig. 2(b)]. The main advantage of representing neuronal ensembles as population vectors is that vectorial analyses could be systematically implemented.^9^^,^^43^ In this way, population metrics could be applied to measure the similarity of neuronal ensembles^27^ at different trials of the behavioral task.^4^ On the other hand, because each population vector captures the relation between all observed neurons, the metrics are independent of the length of the recordings, allowing for the rigorous comparison of the same population of neurons at different times.^9^^,^^43^^,^^48^

A simple but robust visualization of the similarity between population vectors is the implementation of similarity maps.^4^^,^^44^^,^^47^^,^^49^ Similarity maps portray a square matrix that contains the values of all possible combinations of population vectors. An advantage of representing the similarity of population vectors as maps is that the similarity map can grow according to the length of the recordings, providing new information at different times but keeping the previous metrics unaltered.

The goal of neuronal ensemble identification for interventional experiments is to find groups of neurons with coordinated activity that repeat at different times and that have a causal relation to learned behaviors.^48^ It has been shown that similarity maps highlighting groups of neurons with coordinated activity at different times can be factorized using singular value decomposition (SVD) allowing for the identification of neuronal ensembles that are relevant to behavioral performance.^4^ SVD is commonly used to decompose matrices into latent variables that represent repetitive patterns.^50^ In the case of recordings from the primary visual cortex, it has been shown that each factor extracted from SVD represents a neuronal ensemble that was active when a different orientation of drifting-gratings was shown to awake mice.^4^^,^^45^^,^^51^

Recently, different algorithms from calcium imaging recordings have been used to study population activity in mice^4^^–^^8^^,^^14^^,^^45^^,^^46^^,^^51^^–^^57^ (Table 1). The comparison of a subset of such algorithms suggested that a graphical approach that leverages community structure represents the most efficient algorithm to recover neuronal ensembles from simulated and experimental data.^27^ However, such a graphical approach has not been tested for interventional experiments aiming to modulate behavior in mice, making it difficult to summarize which algorithm is better and why. On the other hand, dimensionality reduction algorithms usually have been applied in neuroscience to infer latent variables, to define neuronal population trajectories, or for exploratory analyses;^58^ however, such techniques have not been used to identify and target neurons related to learned behaviors in mice.

**Table 1:** Algorithms used for calcium imaging population analyses in mice: principal component analysis (PCA), pairwise correlations, averaged activity of images, t-distributed stochastic neighbor embedding (t-SNE), locally linear embedding (LLE), singular value decomposition (SVD), similarity graph clustering (SGC), conditional random fields (CRFs), Laplacian eigenmaps.

Algorithm	Input data	Output data	Validation	References
PCA based	Single neurons	Trajectories, ensembles	Shuffled datasets, surrogate data	27, 54
Correlation	Single neurons	Ensembles	Shuffled datasets	14, 56
Average activity	Single neurons	Ensembles	Binary classifiers, sorting data	5–8
t-SNE	Population vectors	Ensembles	Shuffled datasets	53, 57
LLE	Population vectors	Ensembles	Shuffled datasets	55
SVD	Population vectors	Ensembles	Similarity functions	4, 45, 51
SGC	Population vectors	Ensembles	Surrogate data	27
CRFs	Population vectors	Ensembles	ROC curves	4, 46
Laplacian eigenmaps	Population vectors	Trajectories, ensembles	Supervised decoders	52

## 4 Targeting of Neuronal Ensemble Members that Could Influence Learned Behaviors

Interventional experiments have used two different approaches to recall neuronal ensembles related to learned behaviors.

On the one hand, a probabilistic graphical model was used to identify neurons with pattern completion capability that can recall neuronal ensembles associated with the correct performance of the learned behavior.^4^ In such graphs, nodes represent neurons, and edges represent functional connections. In this way, graphs express the conditional interaction between neuronal ensemble elements. Probabilistic graphical models not only capture the functional structure of neuronal ensembles but also highlight the role of individual neurons in each experimental condition. Graphical models could systematically measure the changes in functional connectivity due to learning or optogenetic manipulation.^46^ It was demonstrated that the targeted activation of neurons with pattern completion capability was able to recall neuronal ensembles related to the correct execution of the learned task, improving behavioral performance or evoking the behavioral outcome even in the absence of sensory stimuli.^4^

On the other hand, a different approach was the selection of targeted neurons based on the averaged activity of all of the recorded neurons in the trials related to the learned task.^5^^–^^8^ It was demonstrated that the simultaneous activation of 10 to 30 neurons was able to recruit neuronal ensembles related to the learned behavior.

Both approaches relied on the identification of specific groups of neurons from calcium imaging recordings and the activation of selected neurons using two-photon optogenetics. These studies suggest that the optogenetic activation of a handful group of neurons is sufficient for triggering widespread neuronal ensembles that can modulate behavioral performance.

## 5 Restrictions and Considerations for the Manipulation of Neuronal Ensembles Related to Behavior

The causal relation between neuronal ensembles and learned behaviors has been demonstrated recently in different brain areas.^4^^–^^8^ In this review, we provided a conceptual framework tailored for interventional experiments in behaving mice using simultaneous two-photon calcium imaging and two-photon optogenetics.

The conceptual framework to identify and manipulate neuronal ensembles proposed in this review was implemented for the analysis of calcium transients that represent bursting activity disregarding spike rates. It has been extensively shown that spike rates from electrophysiological recordings provide detailed information of brain computations. However, the technology to control single action potentials in many neurons simultaneously is still under development, so interventional experiments until now have not used single spike synchronization to drive learned behaviors.^4^^–^^6^

Analyses on raw calcium transients to identify neuronal ensembles related to behavior lack biological interpretability because correlations on raw calcium transients introduce artifacts due to the slow decaying phase of calcium fluorescence.^9^^,^^14^^,^^33^

It is important to highlight that neuronal ensemble analyses related to behavior should be validated by controlled experimental conditions; in other words, for “x” controlled experimental conditions, there should be at least “x” neuronal ensembles that match accordingly each experimental condition.^27^^,^^48^ Different approaches could be used to measure if the classification of ensembles is correct, from decoding algorithms^5^^,^^46^ to the use of synthetic data that preserves the statistical properties of experimental data.^27^ However, the strongest argument available for the correct classification of ensembles in interventional experiments is the fact that the reactivation of targeted ensembles related to a learned behavior can evoke such behavior and that the targeting of different ensembles cannot evoke the learned behavior. In this way, even though putative neuronal ensembles could be identified for different brain states, the ultimate proof that a neuronal ensemble is relevant for a learned behavior requires the precise reactivation of such an ensemble with high spatial resolution.

On the other hand, performing independent analyses on the same datasets could be useful for validating the identification of neuronal ensembles. For example, after the identification of neuronal ensembles using any method on population vectors, the correlation between the neurons that belong to a defined neuronal ensemble could be used to corroborate that the neurons identified as an ensemble indeed have coordinated activity,^4^^,^^45^ such corroboration does not represent a circular argument because the identification of neuronal ensembles on population vectors is based on frame similarity that is independent of pairwise correlations between neurons.^27^

It is worth mentioning that two-photon optogenetics can produce spurious activation of nontargeted neurons within a radius of ∼40  μm,^2^^,^^3^^,^^21^^,^^45^ due to movement artifacts or anatomical overlapping. However, it has been demonstrated that the stimulation of randomly selected neurons, in areas where neuronal ensembles are sparsely distributed, rarely coactivate other neurons,^4^^,^^5^^,^^45^ suggesting that off-target activation of neurons would not have a significant effect on the identity of behaviorally relevant ensembles.

Despite that several laboratories can record and manipulate neuronal populations simultaneously, the approaches to identifying which neurons should be targeted are heterogeneous among research groups. The conceptual framework proposed here for the identification and manipulation of neuronal ensembles in behaving mice could provide a first step to standardizing metrics across laboratories and experimental conditions.

The next generation of interventional experiments should consider not only the recalling of ensembles at behavioral time scales^59^ but also the sequential activation of different ensembles that could be related to different stages of the behavioral task in the study.^1^ Conceptually, each ensemble could be represented as a node in a graph, and transitions between ensembles could be represented as edges.^44^^,^^49^^,^^51^

Finally, it has been proposed that the alteration of neuronal ensembles could be related to movement deficits,^55^^,^^60^ memory impairments,^16^^,^^61^^,^^62^ or perceptual deficits,^54^ suggesting that the conceptual framework proposed here could be used to identify neuronal ensembles in different brain regions and create stimulation protocols to revert neuropathological conditions, allowing for the precise reprograming of awry neural microcircuits with single cell resolution.
